# Vitamin D Status Assessment: Lack of Correlation between Serum and Hair 25-Hydroxycholecalciferol Levels in Healthy Young Adults

**DOI:** 10.3390/diagnostics12051229

**Published:** 2022-05-14

**Authors:** Zsolt Gáll, Brigitta Csukor, Melinda Urkon, Lénárd Farczádi, Melinda Kolcsár

**Affiliations:** 1 Department of Pharmacology and Clinical Pharmacy, George Emil Palade University of Medicine, Pharmacy, Science, and Technology of Targu Mures, 540142 Targu Mures, Romania; urkonmelinda1@gmail.com (M.U.); melinda.kolcsar@umfst.ro (M.K.); 2 Faculty of Pharmacy, George Emil Palade University of Medicine, Pharmacy, Science, and Technology of Targu Mures, 540142 Targu Mures, Romania; csukorbrigitta@gmail.com; 3 Chromatography and Mass Spectrometry Laboratory, Center for Advanced Medical and Pharmaceutical Research, George Emil Palade University of Medicine, Pharmacy, Science, and Technology of Targu Mures, 540142 Targu Mures, Romania; lenard.farczadi@umfst.ro

**Keywords:** vitamin D3, 25-hydroxy-vitamin D3, calcidiol, serum concentration, hair concentration, vitamin D status assessment

## Abstract

Vitamin D deficiency has been linked to numerous health problems, including those resulting from disturbed calcium-phosphorus homeostasis, and neuropsychiatric and autoimmune disorders. Nearly one-third of the global population has suboptimal levels of vitamin D, according to epidemiological data. Vitamin D status is usually determined by measuring serum 25(OH)D, but, for decades, serum 25(OH)D measurement has been hampered by a lack of standardization. There have been many recent initiatives to develop reference substances and methods for measuring vitamin D and its metabolites, and re-evaluating the optimal values. It was also suggested that alternative biological samples could also be used, such as hair, since it has been established that lipophilic substances, such as corticosteroids, can also be found in hair. The purpose of this study was to determine the correlation between 25(OH)D3 concentrations in serum and hair, and other demographic features in 26 healthy Caucasian young adult volunteers. The determination of 25(OH)D3 and cholecalciferol was carried out using liquid chromatography coupled with mass spectrometry (LC-MS) from blood and hair samples taken at two timepoints separated by nine weeks. In the hair samples of 18 out of 26 subjects, 25(OH)D was detected at a mean (±SEM) concentration of 17.07 ± 5.375 pg/mg at the first sampling time, and 58.90 ± 25.97 pg/mg at the second sampling time. A multiple linear regression analysis revealed no effects of gender, body mass index, supplementation, or sun exposure on hair 25(OH)D3 concentrations, but supplementation and sun exposure significantly increased serum 25(OH)D3 concentrations. In addition, serum and hair 25(OH)D3 concentrations did not correlate; however, there was a strong correlation between the two sampling times for serum 25(OH)D3 concentrations. In conclusion, this study confirmed that 25(OH)D3 could be detected in human hair, but its use as a biomarker warrants further investigations since no link was found between serum 25(OH)D3 concentrations, supplementation, sun exposure, and hair 25(OH)D3 concentrations levels.

## 1. Introduction

Vitamin D belongs to the secosteroid family, which contains structural similarities with endogenous steroid products (e.g., cortisol and testosterone), with the exception of a bond (C9–C10) being broken in ring B. In its biologically active form, 1,25-dihydroxyvitamin D is referred to as a steroid hormone since it binds and activates the nuclear vitamin D receptor [1,2]. The specific form of vitamin D found in humans is vitamin D3, which is derived from dehydrocholesterol in the skin, where dehydrocholesterol is converted into previtamin D3 in the presence of sunlight. As previtamin undergoes temperature-dependent isomerization, it becomes vitamin D3 and is transported to the liver, where it is converted to the major circulating form, 25-hydroxyvitamin D3 (25(OH)D3), by cytochrome P450 (CYP) 2R1. Several alternative activation pathways of vitamin D3 were discovered in the past decades, involving, along with 25-hydroxylases (CYP27A1, CYP2J2/3, CYP3A4, CYP2D25, and CYP2C11), CYP11A1, which was shown to hydroxylate the side chain of vitamin D3 [3,4]. 25(OH)D3 is then metabolized to 1,25-dihydroxy vitamin D3 (1,25(OH)2D3) within the kidney by cytochrome P450 27B1 [1,5]. Vitamin D is essential to prevent rickets and osteomalacia and it might have a role in fracture repair. Together with adequate calcium intake, it can help to prevent fractures in elderly vitamin-D-deficient individuals [6]. Recent evidence has linked vitamin D deficiency with cancer and metabolic and neurodegenerative diseases, but its causal role remains elusive [7,8,9,10,11,12]. Currently, the normal range for serum 25(OH)D3 is considered to be 20–50 ng/mL (50–125 nmol/L) while less than 12 ng/mL (30 nmol/L) was related to an increased risk of developing rickets/osteomalacia [13]. On the other hand, to manifest vitamin D toxicity, one must have a serum 25(OH)D concentration >150 ng/mL (375 nmol/L) in combination with high calcium consumption [14]. While vitamin D toxicity is rare, vitamin D deficiency is a pandemic that affects over 1 billion people worldwide [15,16,17,18].

Vitamin D deficiency is commonly caused by a lack of sun exposure, which can be brought on by various factors. Although it has been claimed that 15–20 min of sunlight per day on the face and hands is enough to meet daily vitamin D requirements for most skin types and latitudes [19], it has been estimated that ~40% of people living in Europe are vitamin D deficient, especially during the winter [20]. On the other hand, the prevalence of vitamin D deficiency and rickets is high in the Middle East despite abundant sunshine [21]. Other risk factors, including air pollution, the use of sunscreens, shading of high-rise buildings, modern lifestyle, and cultural practices, have been suggested [22]. Moreover, vitamin D deficiency is not uncommon in various forms of chronic kidney disease [23,24]. Considering the increasing prevalence of vitamin D deficiency worldwide, it is now more important than ever to have access to clinical tools for vitamin D assessment.

It has long been known that the serum level of 25(OH)D is an excellent marker of vitamin D status. Due to the stability and long half-lives of both 25(OH)D2 and 25(OH)D3, they are usually quantitated together [25,26,27]. Currently, the most common techniques for measuring 25(OH)D3 are immunoassays, high-performance liquid chromatography (HPLC), and liquid chromatography tandem mass spectrometry (LC–MS/MS) [28,29,30]. However, there are several challenges associated with the analysis of 25(OH)D3 from blood samples. First, the presence of many vitamin D3 derivatives can interfere with the analytical measurement due to their structural similarity. Indeed, one of the main drawbacks of immunoassays is the antibodies’ poor specificity which leads to overestimation of vitamin D metabolites [26]. Second, 25(OH)D3 quantitation is challenging to achieve in aqueous media due to its hydrophobicity. Hence, accurate quantification requires the extraction of 25(OH)D3 correctly, which most commonly includes liquid-liquid extraction (LLE) and solid-phase extraction (SPE) techniques [31]. The third problem with 25(OH)D3 analysis is that 88% of total serum 25(OH)D3 is bound to vitamin-D-binding protein (DBP) while less than 1% is in the free form, with the remaining part being transported by albumin throughout the body [32,33]. In the last decade, notable improvements have been achieved and there are methods available to simultaneously quantify 10 forms of vitamin D in blood [34].

Increasingly sensitive analytical instruments allow the analysis of alternative biological samples, and future developments may result in an innovative approach to the analysis of these metabolites in other body matrices, such as cerebrospinal fluid or hair [35,36]. A recent proof-of-concept study showed that 25(OH)D3 can be extracted from hair samples similarly to cortisol [36]. Furthermore, in a relatively small population pilot study, it was established that a hair-based assay could provide 3-month average levels of 25(OH)D3 [37]. The aim of this study was to evaluate the 25(OH)D3 and cholecalciferol levels in healthy young adults using blood and hair samples and to study the correlation between serum and hair levels. Additionally, the influence of lifestyle factors and supplementation on hair 25(OH)D3 content was also assessed.

## 2. Materials and Methods

### 2.1. Subjects and Sampling

For the current study, 26 (20 female and 6 male) healthy Caucasian young adult volunteers (age 20–35 years, mean ± SD body mass index (BMI) of 21.9 ± 2.16 kg/m^2^) from Targu Mures, Romania were employed in March 2021. This study was conducted according to the guidelines of the Declaration of Helsinki. All volunteers were informed in detail about the procedures and the scope of the investigation and signed consent forms were obtained from all the participants, as per the study protocol approved by the Ethics Committee of George Emil Palade University of Medicine, Pharmacy, Science and Technology of Targu Mures (no. 1298/12.03.2021). The exclusion criteria included hormonal disorders; obesity defined as a BMI greater than 30 kg/m^2^; the use of antiepileptic drugs or anabolic steroids; and chronic cardiovascular and metabolic diseases, such as diabetes mellitus, autoimmune diseases, alopecia, and cancer of any kind. Similarly, dyed hair was also considered to be an exclusion criterion to eliminate confounding factors. Each participant completed a short questionnaire regarding their age, body mass index, physical activity habits, vitamin D3 supplementation, and sun exposure, and were asked to maintain their normal routines during the study. The sample size was calculated using an α (2-tailed) = 0.05, β = 0.25, and an expected correlation coefficient of r = 0.50 [38]. Two sampling points were selected with a three-month gap between them to evaluate the dynamics of changes in serum and hair 25(OH)D3. This design allowed observation of whether the increase/decrease in serum 25(OH)D3 is followed by the same pattern of modification in hair 25(OH)D3 levels. At each timepoint, 6 mL of blood were collected in additive-free tubes via venipuncture and centrifuged within 2 h of collection at 6000 rpm for 10 min at 4 °C (Sigma 2k15, Sigma, Osterode am Harz, Germany). All tubes were kept on ice until processing. Serum samples of 2 mL were collected and stored at −20 °C until analysis. Approximately 100 mg of hair were sampled directly from the vertex posterior of the head regions and kept in labeled and sealed plastic tubes in a cold and dark place before processing.

### 2.2. Standards and Reagents

Standard references for vitamin D3, d3-vitamin D3 (6,19,19-d3) as internal standard, 25(OH)D3, d6-25(OH)D3 (26,26,26,27,27,27-d6) as internal standard, and 1,25(OH)2D3 were purchased from Cerilliant, TX, USA. 4-Phenyl-1,2,4-triazoline-3,5-dione (LiChropur for LC-MS) dissolved in acetonitrile was used for derivatization. All solvents were of high-purity LCMS grade: methanol (ChromaSolv^®^, Sigma-Aldrich, St. Louis, MO, USA), n-hexane (Hipersolv Chromanorm^®^, Sigma-Aldrich, St. Louis, MO, USA), methyl-tert-butyl-ether (ChromaSolv^®^, Sigma-Aldrich, St. Louis, MO, USA), acetonitrile (ChromaSolv^®^, Sigma-Aldrich, St. Louis, MO, USA), and formic acid (Scharlau, Sentmenat, Spain) were used. A Millipore Direct-Q water purification system (Millipore, Bedford, MA, USA) provided the water.

### 2.3. Calibrant and Stock Solutions

To prepare the standard mixtures used as calibrators (working solutions) and internal standards (IS), solutions of 1.0 and 0.5 μg/mL of analytical and labeled standards were prepared in methanol. These solutions were kept at −20 °C and the stability of the analytes was monitored throughout the analysis. As for the concentrations of analytes in the calibration and IS mixtures, these were selected according to their analytical limit of detection (LOD) and expected concentration in serum and hair samples. The calibration curve used for the analysis of serum samples was built to include 6–8 calibration points.

### 2.4. Sample Preparation

Serum samples spiked with internal standards underwent protein precipitation with an equal volume of acetonitrile (350 µL) and the analytes were extracted with a mixture of methyl-tert-butyl-ether and hexane (1:1, *v*/*v*). After testing the extraction method with both methyl-*tert*-butyl-ether and hexane alone, using the mixture resulted in better extraction recovery (50:50; *v*/*v*). The organic layer was separated and then evaporated by vacuum centrifugation at 35 °C for 0.5 h (Thermo Scientific Savant SpeedVac Concentrator, SPD121P, ThermoFisher Scientific, Marietta, OH, USA). The dry residue was reconstituted in 150 μL of acetonitrile, and 50 μL of the 4-phenyl-1,2,4-triazoline-3,5-dione (PTAD) derivatizing reagent (stock solution of PTAD, 2 mg/mL) were added followed by30 s of vortexing. The mixture was protected from light and left to stand at room temperature overnight.

Hair samples were washed in 1 mL of isopropanol for 3 min at room temperature to remove contaminants, such as sweat, sebum, and hair care products, from the outer surface of the hair. Samples were then dried on paper towels and cut into shorter strips. In total, 50 mg of hair sample were weighted using an analytical scale (Mettler Toledo, AB 54-S, Mettler Toledo, Greifensee, Switzerland), transferred into a glass tube, and the analytes of interests were extracted by adding 3 mL of methanol and vortexing for 24 h using an orbital shaker (Multi Reax, Heidolph Instruments, Schwabach, Germany). After the extraction period, samples were centrifuged at 1300× *g* for 15 min (Eppendorf centrifuge 5430R, Eppendorf AG, Hamburg, Germany) and 2 mL of supernatant were transferred into Eppendorf tubes and then evaporated by vacuum centrifugation at 35 °C for 2 h (Thermo Scientific Savant SpeedVac Concentrator, SPD121P, ThermoFisher Scientific, Marietta, OH, USA). Samples were resuspended in 150 µL of acetonitrile followed by the addition of 50 µL of derivatizing reagent (stock solution of PTAD, 2 mg/mL). The mixture was vortexed for 30 s and left to react overnight at room temperature in a dark place.

The 25(OH)D3 and cholecalciferol content of the hair samples was calculated using the following formula: C (pg/mg) = [measured concentration (ng/mL) × volume of extraction (mL)] × 1000/[volume of evaporated/reconstituted samples (ml) × weight of hair sample (mg)], similarly to Zgaga et al. [36].

### 2.5. LC-MS/MS System

**Figure 1:**
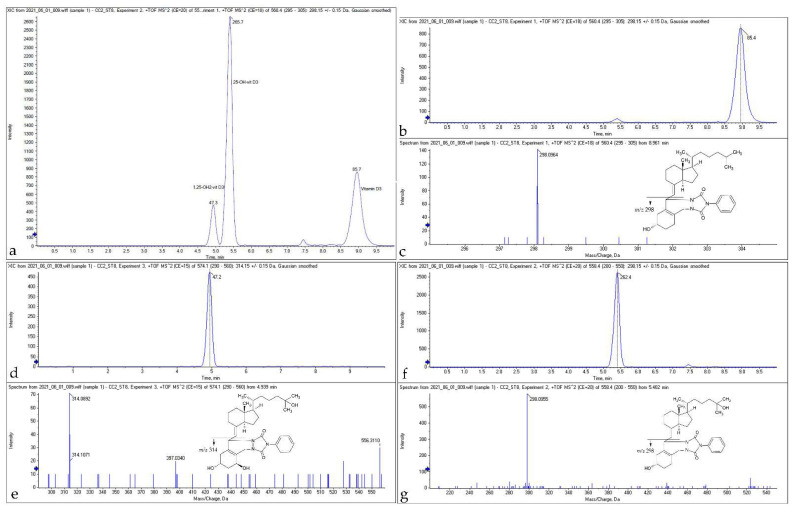
Representative merged chromatograms (**a**) and mass spectra of serum samples spiked with standard solutions of vitamin D3 (**b**,**c**), 25(OH)D3 (**f**,**g**), and 1,25(OH)2D3 (**d**,**e**), containing 50 ng/mL analyte extracted by liquid-liquid extraction using a mixture of methyl-tert-buthyl ether and hexane (1:1) and analyzed with UHPLC-ESI-MS/MS after derivatization with PTAD.

LC MS/MS analysis was performed using a Flexar FX10 (Perkin Elmer, Waltham, MA, USA) ultra-high-performance liquid chromatographic system coupled with a QTOF 4600 mass spectrometer (AB Sciex, Concord, CA, USA). Data acquisition and analyses were performed using Analyst TF software version 1.7.1 (AB Sciex Concord, ON, Canada). Chromatographic separation of the analytes of interest was performed on a Phenomenex Gemini 3 µm NX-C18 110 Å column. The mobile phase consisted of a mixture of 90% water containing 0.1% formic acid and 10% acetonitrile (eluent A) and methanol (eluent B) and the flow rate was set at 0.5 mL/min and an oven temperature of 25 °C. The following gradient elution was used: 60% eluent B for 2.0 min; 60–90% B from 2.0 to 4.5 min; and held at 90% from 4.5 to 11.0. From 11.0 to 12.0 min, the column was re-equilibrated to 60% B. The autosampler was cooled at 20 °C. The injection volume was 50 µL. A representative chromatogram of a calibrator containing vitamin D3, 25(OH)D3, and 1,25(OH)2D3 along with the mass spectra of each analyte is shown in Figure 1.

The detection parameters were optimized for each analyte. Ionization was performed in electrospray positive mode and the source parameters were set as follows: ionspray voltage floating: 4300 V; vaporizer temperature: 600 °C; ion source gas 1: 32; ion source gas 2: 25; curtain gas: 25; ion release delay: 30; ion release width: 15. The MRM parameters (retention time, precursor ion/product ion, declustering potential, collision energy) for all analytes and the corresponding internal standard (IS) are presented in Supplementary Table S1.

### 2.6. Method Validation

The method was validated using spiked blank matrices prepared as described above for the preparation of the samples. As part of the method validation, the quality control samples (*n* = 5 each of QCA, QCB, and QCC) were measured. QC samples were prepared following the same procedure serum as the calibrators. A total of five QC samples (*n* = 5) of each concentration level were analyzed on the same day to calculate the intraday precision and accuracy. Three successive analytical runs were used to determine the interday precision and accuracy. Precision was calculated as the coefficient of variance (CV) of the intraday and interday analytical results. Accuracy was determined as the recovery of each analyte in the QC samples at three levels. The accuracy of each analyte was assessed by comparing the QC samples with the average levels of mixed blank samples with the standard added. Results were analyzed using the peak area ratio between the analyte and the corresponding IS. Calibration curves were generated by subtracting the endogenous amount of an analyte from the spiked amount (blank subtraction). The results of the analysis allowed the determination of LOD, LLOQ, accuracy, linearity, precision, and stability. The effect of the matrix on the analytes was also assessed. The increase in the peak area ratios of the analytes was compared with the respective area ratio measured in the calibrator solutions to which the same levels of standards were added.

### 2.7. Statistical Analysis

Data acquisition was operated by Analyst TF 1.7.1 software (AB Sciex, Framingham, MA, USA). Microsoft Excel was used to perform statistical analysis of the method validation results, including calculation of the mean, the standard deviation, and the coefficient of variance. The least-squares method was used in linear regression analysis to evaluate the calibration curve for each analyte. Associations between analytes in hair and serum were assessed using the Pearson correlation coefficient. Multiple linear regression was used to determine which specific parameters contribute to 25(OH)D3 levels in serum and hair. The significance level was set a priori at α ≤ 0.05.

## 3. Results

### 3.1. Method Development and Validation

The method was validated for vitamin D3 and 25(OH)D3 using spiked serum samples, and the interday and intraday precision and accuracy values and recovery values, which were calculated and are summarized in Table 1. The LLOQ value was defined as the concentration at which the signal-to-noise ratio was 10 and there were positive values after subtracting the blank values from the spiked samples (the area ratio of the analyte in spiked samples subtracting the area ratio of the analyte in blank matrix). For the analytes not detected in the matrix, LOD and LLOQ were determined as explained above for LLOQ of the endogenous substances (signal-to-noise ratio 3/10 for LOD/LLOQ). The lower limit of detection (LOD) was found to be 1 ng/mL and 5 pg/mg with a linear range from 2 to 50 ng/mL in serum and 15 to 2000 pg/mg in hair for both analytes, respectively. The linearity was determined by making sure that the linear regression (R^2^) values for the calibration curve were equal to or greater than 0.99. The calibration curve was accepted with at least six calibrants on the curve, according to international guidelines. Brief cross-validation was also performed on the hair samples.

### 3.2. Vitamin D Status of the Volunteers

The quantitative determination of the 25(OH)D3 and vitamin D3 concentrations in serum and hair samples and the statistical evaluation of these results are summarized in Table 2. Although the serum 25(OH)D3 levels of subjects were significantly lower in March (14.11 ± 1.259 ng/mL) than in May (17.67 ± 1.342 ng/mL; *p* = 0.0013, paired *t* test), the correlation between the two sampling points was highly significant (r = 0.717, *p* < 0.0001). The serum concentration of vitamin D3 (median (range) 0.175 (0–16.64) vs. 0.9 (0–12.85), W = 30, *p* = 0.212, Wilcoxon test) did not significantly differ between the two sampling points, but a significant correlation between the two sampling points was noted (Spearman r = 0.467, *p* = 0.016) (Table 2). As for the 25(OH)D3 levels in hair samples, there was neither a significant difference nor correlation with the sampling time.

### 3.3. The Correlation between Demographic and Lifestyle and Vitamin D3 Metabolites from Serum and Hair Samples

A multiple linear regression model that incorporated gender, BMI, vitamin D3 supplementation, intentional sun exposure, and regular physical activity habits of the volunteers was constructed to analyze the influence of these factors on 25(OH)D3 concentrations measured in serum (Supplementary Tables S2 and S3). The results indicated that intentional sun exposure (|t| = 2.294, *p* = 0.0328) at t2 and vitamin D3 supplementation (|t| = 2.104, *p* = 0.0482) at t1 were related to 25(OH)D3 concentrations in serum.

**Figure 2:**
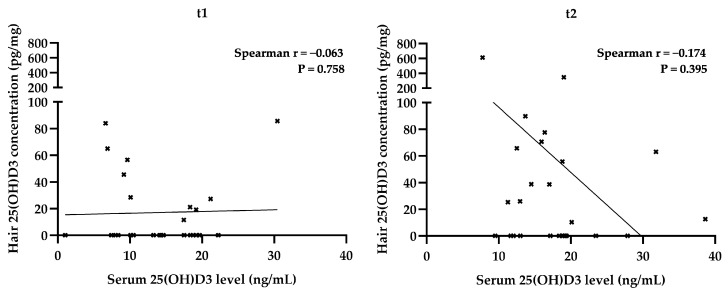
Correlation of hair and serum 25(OH)D3 levels of 26 healthy Caucasian adult subjects whose blood and hair were sampled at 2 different timepoints t1 and t2. The values of Spearman’s correlation coefficients show that there is no relationship between the variables.

Furthermore, Spearman correlation coefficients were calculated separately for serum vitamin D3 concentrations and hair 25(OH)D3 concentrations because these datasets were non-normally distributed. A statistically significant correlation was found between vitamin D3 supplementation and serum vitamin D3 concentrations (Table 3). However, hair 25(OH)D3 concentrations did not show any correlation with the studied parameters (Figure 2).

**Figure 3:**
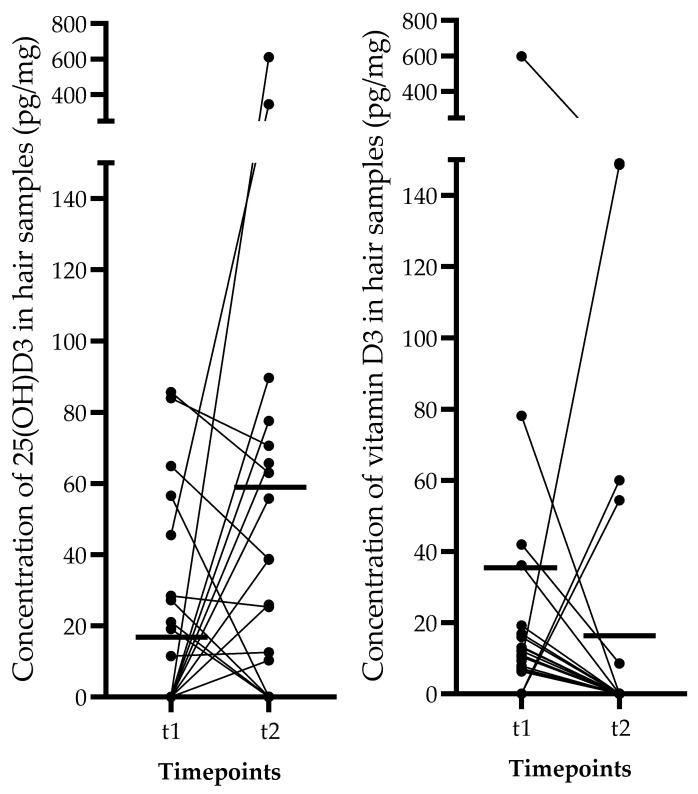
Individual 25(OH)D3 and vitamin D3 levels in the hair of 26 healthy Caucasian adult subject. Only 18 subjects provided hair samples that had detectable (>5 pg/mg) levels of 25(OH)D3. Solid line indicates the mean concentration at the specified timepoint.

After analyzing the variation in the serum concentrations of 25(OH)D3 and vitamin D3 in the subgroup of volunteers taking supplements, it can be observed that higher serum levels of both 25(OH)D3 and vitamin D3 were measured at both t1 and t2 timepoints, but the significance limit was not reached. Conversely, intentional sun exposure did not influence the serum vitamin D3 levels, but it had a significant impact on the serum 25(OH)D3 concentrations (F (1, 22) = 4.876, *p* = 0.038, 2-way ANOVA). However, neither sun exposure nor supplementation had an effect on hair 25(OH)D3 or vitamin D3 concentrations (Figure 3).

## 4. Discussion

As far as the authors are aware, this study is the first attempt to evaluate the correlation between 25(OH)D3 extracted from hair samples and serum samples in healthy Caucasian adult subjects. Summarizing the main findings of this study, one can state that, first, a robust and highly sensitive analytical method has been developed and validated for the quantification of vitamin D3, 25(OH)D3, and 1,25(OH)2D3. Second, more than half of the hair samples collected from healthy volunteers contained 25(OH)D3, which is consistent with literature studies, since Shah et al. identified 25(OH)D3 in 50 out of 70 samples in young males from the United Arab Emirates [37]. Moreover, Zgaga et al. also reported that not all hair samples contained a quantifiable amount of 25(OH)D3 [36]. The amount of 25(OH)D3 found in the hair samples ranged from 11.5 to 611.2 pg/mg in this study, whereas Shah et al. [37] reported an amount that ranged from 10 to 1500 pg/mg and Zgaga et al. [36] found a range of 11.9 to 911 pg/mg. Third, this study reports for the first time that vitamin D3 can also be detected in hair samples, especially in subjects taking vitamin D3-containing supplements.

It is important to note that the vitamin D3 status of the volunteers enrolled in this study had a significant impact regarding vitamin D3 supplementation and intentional sun exposure on serum 25(OH)D3 levels. These findings are in accordance with previous reports that both sun exposure and vitamin D3 supplementation (as low as 600 IU) can raise serum 25(OH)D3 levels in less than 3 months [39,40,41,42]. Furthermore, it is also noted that sun exposure had a stronger influence on serum 25(OH)D3 levels than vitamin D3 supplementation, which is in agreement with the results reported by Bogh et al. [43].

However, no correlation was observed between serum 25(OH)D3 or vitamin D3 and hair 25(OH)D3 concentrations. The use of hair samples as an alternative means of monitoring a patient’s corticosteroid levels has been proposed previously [44,45,46]. It was hypothesized that long-term exposure to cortisol might be reflected in the amount of cortisol in hair. Two major factors should be considered: (1) hair grows slowly, about 1 cm per month, and all steroids and steroid-like compounds from the circulation are incorporated in its matrix; and (2) cortisol is short-lived in the blood, and high variability can be observed throughout the day. The latter fact makes the use of serum cortisol levels as a biomarker of stress or related health conditions difficult. Although hair cortisol has never been correlated with other markers of the hypothalamic-hypophyseal axis, and local cortisol synthesis has been shown to take place in the skin [45,47], a large number of observational studies have found a link between psychiatric disorders and hair cortisol levels. However, in the case of vitamin D3 status, total serum 25(OH)D3 can be considered a stable biomarker of vitamin D supplementation, since according to the findings of all randomized controlled trials, serum 25(OH)D concentration increases in response to treatment with supplemental vitamin D, regardless of whether ergocalciferol or cholecalciferol were used, or the analytical technique or length of study (6 week to >2 years) [48]. Recently, several issues have been raised regarding the use of total serum 25(OH)D3 for diagnosis of vitamin D deficiency. These issues are mainly related to analytical challenges and controversies regarding the interpretation of results, and some authors have recommended the use of different levels of optimal 25(OH)D for various health outcomes, which significantly undermines its reliability [25,49,50]. Nevertheless, the current study results indicate that the use of hair 25(OH)D3 is even more difficult because the same analytical challenges remain unresolved, and other factors are encountered in the sample preparation and extraction phase.

The vitamin D content in hair follicles depends on many factors. Based on the theory of free hormone fraction, the DBP-unattached form of 25(OH)D enters the hair follicles. Although it has not yet been described in scalp hair follicles, the existence of endocytic transporters (megaline, cubilin) can facilitate intracellular entry of the DBP-bound form of vitamin D and its metabolites in different cells (as demonstrated in the kidney, parathyroid, inner ear cells) [51]. Another important fact to note is that local synthesis in the hair follicle (especially in keratinocytes) is also possible for vitamin D3 metabolites, including 25(OH)D3. It is well known that vitamin D3 is mainly produced in the epidermis by converting 7-dehydrocholesterol under the influence of UVB to first form pre-vitamin D3, which, when isomerized, is converted to vitamin D3. However, the epidermis also contains 25-hydroxylase CYP27A1, and possibly CYP2R1, and the 1α-hydroxylase CYP27B1 and the vitamin D receptor [52]. Moreover, recent evidence demonstrated that a novel secosteroid activation pathway, which involves CYP11A1, is present in the skin [3,4,53,54]. As a result of CYP11A1 activity, several monohydroxy metabolites of vitamin D3 can be found along with 25(OH)D3, which may interfere with analytical assays. Especially, 20(OH)D3 was shown to reach higher tissular levels than 25(OH)D3 in the epidermis [3,47]. Hence, the total amount of 25(OH)D3 found in hair does not necessarily reflect systemic levels but rather locally synthesized levels as well. According to the results of this study, 25(OH)D3 levels in hair were not correlated between the 2 sampling points, suggesting that hair 25(OH)D3 concentrations can change in less than 3 months, whereas serum 25(OH)D3 concentrations show a strong correlation with previously determined levels.

There are a number of limitations present in this study that can be improved upon in future research. First, the sample size was proven to be small in the detection of the influence of certain lifestyle factors. Second, the amount of vitamin D3 used for supplementation was not controlled for; thus, high variability in the serum vitamin D concentration between subjects could have had an impact on our observations. Third, sun exposure was not quantified, and only an estimation provided by the subjects was available. Fourth, the LC-MS method employed in this study does not quantify 25(OH)D2 and does not resolve inactive epimers of 25(OH)D3; however, their circulating concentrations are very low in adults and might not have an impact on 25(OH)D3 levels in hair. The uneven gender distribution among the participants could also be a limitation of the present study.

## 5. Conclusions

In conclusion, this study confirmed that 25(OH)D3 could be detected in human hair, but the use of hair 25(OH)D3 levels as a biomarker warrants further investigations since no link was found between serum 25(OH)D3 concentrations, supplementation, sun exposure, and hair 25(OH)D3 concentration levels.

## Figures and Tables

**Table 1:** Method validation results for intraday/interday precision, accuracy, and recovery in human serum samples.

Analytes	Concentration Level	Intraday (*n* = 5)		Interday (*n* = 4)	
		Precision, % CV	Accuracy, %	Precision, % CV	Accuracy, %
25(OH)D3	LLOQ 2 ng/mL	16.98%	96.9%	11.52%	100.5%
	QCA 8 ng/mL	10.84%	114.2%	10.72%	104.1%
	QCB 17.5 ng/mL	3.38%	110.1%	9.87%	100.2%
	QCC 30 ng/mL	3.72%	108.7%	7.49%	108.7%
vitamin D3	LLOQ 2 ng/mL	4.14%	113.2%	4.16%	111.4%
	QCA 8 ng/mL	6.59%	104.4%	4.00%	103.4%
	QCB 17.5 ng/mL	14.49%	100.3%	13.79%	99.9%
	QCC 30 ng/mL	10.74%	107.7%	10.75%	107.2%

**Table 2:** A comparison of the parameters determined during the two sampling periods.

Parameters	Mean ± SEM/Median (Range)		*p* Value
	t1	t2	
Serum 25(OH)D3 (ng/mL)	14.11 ± 1.26	17.67 ± 1.34	0.001 **
Serum vitamin D3 (ng/mL)	0.175 (0–16.64)	0.9 (0–12.85)	0.212
Hair 25(OH)D3 (pg/mg)	0.0 (0–85.63)	11.44 (0–611.2)	0.211
Hair vitamin D3 (pg/mg)	9.86 (0–598.3)	0.0 (0–149)	0.321

The data are expressed as mean ± SEM for normally distributed data, and median (range) for non-gaussian data. ** Significant difference was observed between t1 and t2.

**Table 3:** Correlation between demographical data and the serum and hair concentrations of 25(OH)D3.

Dependent Variable	Timepoint (t)	Independent Variable	Correlation (Spearman *r*)	*p* Value
Serum 25(OH)D3	1	Gender	−0.012	0.953
		BMI	−0.197	0.335
		Supplementation	0.206	0.313
		Sun exposure	0.158	0.440
		Physical activity	0.083	0.686
	2	Gender	−0.049	0.813
		BMI	−0.291	0.149
		Supplementation	0.082	0.689
		**Sun exposure**	**0.390**	**0.049**
		Physical activity	0.189	0.355
		**Serum 25(OH)D3 at t1**	**0.717**	**<0.001**
Serum vitamin D3	1	Gender	−0.039	0.850
		BMI	−0.083	0.687
		**Supplementation**	**0.418**	**0.034**
		Sun exposure	−0.248	0.222
		Physical activity	0.024	0.908
	2	Gender	−0.224	0.272
		BMI	0.133	0.516
		Supplementation	0.273	0.177
		Sun exposure	−0.199	0.329
		Physical activity	0.363	0.068
		**Serum vitamin D3 at t1**	**0.467**	**0.016**
Hair 25(OH)D3	1	Gender	0.056	0.787
		BMI	−0.041	0.844
		Supplementation	0.106	0.607
		Sun exposure	0.253	0.213
		Physical activity	−0.203	0.320
		Serum 25(OH)D3 at t1	−0.063	0.758
	2	Gender	−0.090	0.663
		BMI	−0.058	0.777
		Supplementation	−0.135	0.510
		Sun exposure	−0.272	0.179
		Physical activity	−0.135	0.512
		Serum 25(OH)D3 at t2	−0.174	0.396
		Hair 25(OH)D3 at t1	0.148	0.471

## Data Availability

The data presented in this study are available on request from the corresponding authors.

## Supplementary Materials

The following supporting information can be downloaded at: https://www.mdpi.com/article/10.3390/diagnostics12051229/s1, Table S1: MRM transitions and parameters for vitamin D analysis; Table S2: Multiple regression analysis of the serum 25(OH)D3 levels at the second sampling timepoint (t2); Table S3: Multiple regression analysis of the serum 25(OH)D3 levels at the first sampling timepoint (t1).
