# Supplementary material for: Vitamin D Status Assessment: Lack of Correlation between Serum and Hair 25-Hydroxycholecalciferol Levels in Healthy Young Adults
Source: Diagnostics (Basel). 2022 May 14;12(5):1229. doi: 10.3390/diagnostics12051229 (PMC9140052; doi:10.3390/diagnostics12051229)
Supplement: Supplementary file 1 [file diagnostics-12-01229-s001.zip › diagnostics-1722650-supplementary.pdf]

Supplementary Materials

Vitamin D Status Assessment: Lack of Correlation between Serum and Hair 25-hydroxycholecalciferol Levels in Healthy Young Adults

Zsolt Gáll [1,*], Brigitta Csukor [2], Melinda Urkon [1], Lenard Farczádi [3], and Melinda Kolcsár [1]

[1] Department of Pharmacology and Clinical Pharmacy, George Emil Palade University of Medicine, Pharmacy, Science, and Technology of Targu Mures, 540142 Târgu Mureș, Romania; urkonmelinda1@gmail.com (M.U.); melinda.kolcsar@umfst.ro (M.K.)
[2] Faculty of Pharmacy, George Emil Palade University of Medicine, Pharmacy, Science, and Technology of Targu Mures, 540142 Targu Mures, Romania; csukorbrigitta@gmail.com
[3] Chromatography and Mass Spectrometry Laboratory, Center for Advanced Medical and Pharmaceutical Research, George Emil Palade University of Medicine, Pharmacy, Science, and Technology of Targu Mures, 540142 Targu Mures, Romania; lenard.farczadi@umfst.ro

Table S1. MRM transitions and parameters for vitamin D analysis

Analyte	Retention Time (min)	MRM transition	Collision energy (eV)	Declustering Potential
25(OH)D3	5.40	558.4 – 298.1	20	100
d6-25(OH)D3	5.40	564.4 – 298.1	20	100
vitamin D3	8.95	560.4 – 298.1	18	100
d3-vitamin D3	8.95	563.4 – 301.1	18	100
1,25(OH)2D3	4.95	574.1 – 314.1	15	50

Table S2. Multiple regression analysis of the serum 25(OH)D3 levels at the second sampling timepoint (t2).

Variable	Estimate	Standard error	|t|	P value
Intercept	32.56	16.92	1.924	0.0687
Gender	-2.347	3.571	0.6573	0.5185
Takes supplements?	0.4874	2.432	0.2004	0.8432
Exposed to sun?	5.721	2.494	2.294	0.0328
BMI	-0.8865	0.7123	1.245	0.2277
Physical activity	3.857	2.776	1.390	0.1799

Table S3. Multiple regression analysis of the serum 25(OH)D3 levels at the first sampling timepoint (t1).

Variable	Estimate	Standard error	|t|	P value
Intercept	14.48	17.73	0.8167	0.4237
Gender	0.4714	3.659	0.1288	0.8988
Takes supplements?	5.382	2.558	2.104	0.0482
Exposed to sun?	4.439	2.527	1.757	0.0942
BMI	-0.3728	0.7551	0.4938	0.6268
Physical activity	2.900	2.811	1.032	0.3145
